# In silico and in vitro anti-inflammatory study of phenolic compounds isolated from *Eucalyptus maculata* resin

**DOI:** 10.1038/s41598-023-28221-y

**Published:** 2023-02-06

**Authors:** Dalia E. Ali, Rania A. El Gedaily, Shahira M. Ezzat, Maged A. El Sawy, Meselhy R. Meselhy, Essam Abdel-Sattar

**Affiliations:** 1 Department of Pharmacognosy, Faculty of Pharmacy, Pharos University in Alexandria, Alexandria, Egypt; 2 Department of Pharmacognosy, Faculty of Pharmacy, Cairo University, El-Kasr El-Aini St, Cairo, 11562 Egypt; 3 Department of Pharmacognosy, Faculty of Pharmacy, October University for Modern Science and Arts (MSA), 6th October, 12451 Egypt; 4 Department of Pharmaceutical Chemistry, Faculty of Pharmacy, Pharos University in Alexandria, Alexandria, Egypt

**Keywords:** Biological models, Isolation, separation and purification, Structure determination, Drug discovery, Biomarkers

## Abstract

Plant resins are rich in bioactive compounds with high medicinal values. However, the chemistry and anti-inflammatory activity of the resins produced by trees of the genus *Eucalyptus* were scarcely investigated. The inflammatory targets cyclooxygenase-1 (COX-1), COX-2, TNF-, NF-B, and NO were significantly inhibited by the methanolic extract of *Eucalyptus maculata* kino resin (EME) and its CH_2_Cl_2_ soluble fraction (MCF). Sakuranetin (**C1**), (*E*)-cinnamic acid (**C2**), kaempferol 7- methyl ether (**C3**), 7-*O*-methyl aromadendrin (**C4**), and 1,6- dicinnamoyl-*O-α*-D-glucopyranoside (**C5**) were isolated from MCF. Three compounds (**C1**, **C2,** and **C4**) showed potent in vitro COX-1 inhibition, while **C5** inhibited COX-2, TNF-*α*, NF-κB, and NO significantly. An in-silico study revealed that **C5** had the highest binding affinity to the active site in COX-2 with binding energy score (S) of -14.85 kcal/mol, better than celecoxib (COX-2 inhibitor). In conclusion, 1,6-dicinnamoyl-*O-α*-D-glucopyranoside (**C5**) could be investigated further in the search for anti-inflammatory agents.

## Introduction

Inflammation is a complex, dynamic response to cell injury, infection, trauma, or toxins that can last for a few days (acute inflammation) or for a longer duration (chronic inflammation). The overproduction of pro-inflammatory cytokines, such as tumor necrosis factor-*α* (TNF-*α*), interleukin-1b (IL-1b), and IL-6, as well as inflammatory targets, secreted by immune cells and macrophages, all play an important role in mediating the inflammatory reactions^1^. Also, there is a close link between inflammation and oxidative stress, as one activates the other^2^. Moreover, inflammation triggered by oxidative stress can cause various ailments, such as cancer, rheumatoid arthritis, asthma, and diabetes.^3^ Several polyphenols are known to exert anti-inflammatory effects through modulation of different signaling pathways such as arachidonic acid (AA) metabolism, nuclear factor-kappa B (NF_K_B), and TNF-*α*^4^. In the AA-dependent pathway, the anti-inflammatory effect of plant polyphenols is related to their ability to inhibit cyclooxygenase (COX), which converts AA into prostaglandins.

Medicinal plants are considered a valuable source of potential anti-inflammatory agents^5^. Evidence to support this anti-inflammatory effect is lacking, as most of these plants have not been subjected to chemical, pharmacological, or toxicological studies in order to investigate their bioactive compounds^6^. *Eucalyptus* is one of the most important genera in the family *Myrtaceae*, which includes 132 genera and 5950 species. *Eucalyptus maculata* Hook. is indigenous to Australia and is cultivated in Egypt. *E. maculata* is an evergreen tree that reaches up to 60 m in height, with fragrant white flowers and small brown to green fruit. It has smooth mottled pinkish grey or bluish grey, often dimpled bark that is shed in small, irregular flakes^7^. *Eucalyptus* kino resin, also known as "blood gum," has a very dark color like blood and is exuded by eucalyptus trees (*Angophora Corymbia*, and *Eucalyptus* spp.). It is known to contain high levels of potentially useful polyphenolic compounds^8,9^. Previous work on *Eucalyptus* kino resin resulted in the isolation of (*E*)-cinnamic acid, sakuranetin, 7-*O*-methyl aromadendrin, and 1,6-dicinnamoyl-*O-α*-d-glucopyranoside^9^, in addition to *p*-coumaric acid, 1-*O*-cinnamoyl 6-*O*-*p-*coumaroylglucose, and 7-methyl-aromadendrin-4′-*O*-(6′′-*trans-p-*coumaroyl)-β-D-glucopyranoside^8^. Also, the methanolic extract of the kino resin (EME), had previously demonstrated promising in vivo anti-inflammatory and in vitro antioxidant activities^10^. Sakuranetin, 7-*O*-methyl aromadendrin, and 1,6-dicinnamoyl-*O*-*α* -D-glucopyranoside showed antioxidant and hepatoprotective effects^11^. 7-*O*-methyl aromadendrin had cytotoxicity against MCF-7 and C32 cell lines^12^, it stimulated glucose uptake and improved insulin resistance^13^. Sakuranetin also possessed antioxidant, antibacterial, anti-inflammatory, antiparasitic, antimutagenic, and antiallergic characteristics, and it exhibited antiviral activity against human rhinovirus 3 and influenza B virus^14^. These findings prompted the authors to further fractionate EME to identify and isolate the active anti-inflammatory agent(s) from the kino resin. Furthermore, a molecular docking study was performed to study the binding mode of the active compounds to the active site of COX-2.

## Results and discussion

### Identification of the bioactive compounds from *E. maculata* exudate.

**Figure 1:**
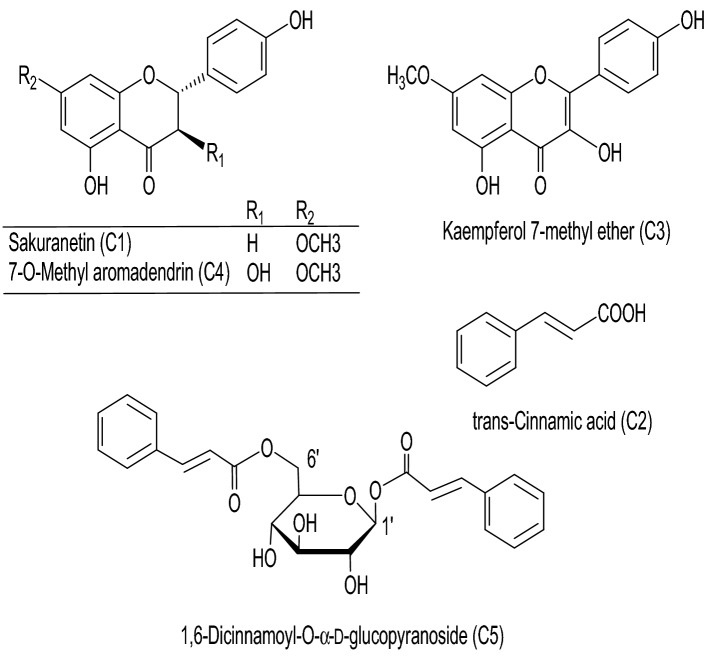
Chemical structures of compounds isolated from *E. maculata* resin.

Five compounds (**C1**–**C5**) (Fig. 1) were isolated from the bioactive methylene chloride fraction of *E. maculata*. The isolated compounds were identified by TLC comparison with the previously isolated compounds or reference standards, spectroscopic analysis (Suppl. Data S1), and comparison with the reported data in literature as sakuranetin [(*2S*)-5,4'-dihydroxy-7-methoxyflavanone] (**C1**)^9,15^, (*E*)-cinnamic acid (**C2**)^9,16^, kaempferol 7- methyl ether (**C3**) (isolated for the first time from MEEM^15,17^), 7-*O*-methyl aromadendrin (**C4**)^9,15^, and 1,6-dicinnamoyl-*O-α*-D-glucopyranoside (**C5**)^9^ (Fig. 1). The *trans*-form of **C2** was deduced from the large coupling constant of the olefinic protons with *J* = 16 Hz of H*α*, β.

### In vitro anti-inflammatory activity of selected isolated compounds

#### The effect on COX-1 and COX-2

Aspirin is one of the most famous non-steroidal anti-inflammatory drugs. It carries out its anti-inflammatory effect through the rough inhibition of two enzymes: COX-1 and COX-2. Its action is associated with gastric bleeding, mainly due to the inhibition of COX-1. So, our main target was the development of more selective COX-2 inhibitors like celecoxib (celebrex®)^18^. COX-1 and COX-2 were estimated according to the reported method^19^ and were compared to indomethacin and celecoxib (selective COX-2 and selective COX-1 inhibitors, respectively). The 50% inhibitory concentration (IC_50_) values were calculated using the concentration-inhibition response curve and were presented as mean ± standard deviation (SD). All enzyme inhibition activities were performed in triplicate and were examined using the sample t-test using the SPSS 26.0 program at (*p* < 0.05) (Table 1).

**Table 1:** COX-1, COX-2, NF-_*K*_B and NO inhibitory activity and TNF-R2 binding activity (IC_50_) of EME, MCF and selected isolated compounds.

Extract/isolated compounds	COX-1* IC_50_ ± SD (µg/mL)	COX-2* IC_50_ ± SD (µg/mL)	TNF–R2* IC_50_ ± SD (ng/mL)	NF- _K_B* IC_50_ ± SD (Pg/mL)	NO* IC_50_ ± SD (µg/mL)
Indomethacin (standard for COX-1)	0.23 ± 0.00	–	–	–	–
Celecoxib (standard for COX-2)	…….	1.20 ± 0.00	–	–	–
Certolizumab	–	–	6.70 ± 0.07	–	–
Curcumin	–	–	–	7.80 ± 0.03	–
7- nitroindazole	–	–	–	–	49.20 ± 0.80
Methanolic extract of *E. maculata* (EME)	0.27 ± 0.01**	1.37 ± 0.06 **	4.83 ± 0.03**	5.77 ± 0.07 **	33.20 ± 0.80**
Methylene chloride fraction (MCF)	0.24 ± 0.01	1.86 ± 0.03 **	5.87 ± 0.09 **	6.73 ± 0.09 **	42.00 ± 2.50
Sakuranetin (**C1**)	0.19 ± 0.01**	3.23 ± 0.01**	8.87 ± 0.09 **	10.07 ± 0.09 **	82.00 ± 1.06 **
Cinnamic acid (**C2**)	0.16 ± 0.10**	5.13 ± 0.01**	7.63 ± 0.03 **	8.77 ± 0.07 **	66.00 ± 0.69 **
7-*O*-methyl aromadendrin (**C4**)	0.22 ± 0.01	2.55 ± 0.02**	8.33 ± 0.09 **	9.33 ± 0.03 **	76.40 ± 1.74 **
1,6- dicinnamoyl-*O-α*-D-glucopyranoside (**C5**)	0.30 ± 0.01**	1.16 ± 0.01**	5.17 ± 0.07 **	6.07 ± 0.07 **	38.00 ± 0.04 **

*Average of three determinations.

(**) Significantly different (P < 0.05) compared to reference drug using sample t-test and using the SPSS 26.0 software.

(*E*)-Cinnamic acid **(C2)** and sakuranetin **(C1)** exhibited remarkable, significant inhibition of COX-1 with IC_50_ of 0.16 ± 0.10 and 0.19 ± 0.01 respectively, meanwhile, Indomethacin (IC_50_ 0.23 ± 0.00 µg/mL) was not statistically different from 7-*O*-methyl aromadendrin **(C4)** (IC_50_ of 0.22 ± 0.01 µg/mL) at (*p* < 0.05). Zhang et al.^15^ previously reported that sakuranetin and 7-*O*-methyl aromadendrin possessed moderate inhibitory effects on COX-1.

Concerning COX-2 inhibitory activity, 1,6-dicinnamoyl-*O-α*-D-glucopyranoside **(C5)** showed the most significant effect with IC_50_ of 1.16 ± 0.01 µg/mL, compared to celecoxib (IC_50_ 1.2 ± 0.00 µg/mL) at (*p* < 0.05). In addition, 7-*O*-methyl aromadendrin **(C4)** and sakuranetin **(C1)** showed moderate inhibitory activity towards COX-2. Sakuranetin was previously reported to suppress the synthesis of COX-2^20^. In contrast, Zhang et.al (2006)^15^ stated that 7-*O*-methyl aromadendrin and sakuranetin had no inhibitory activity against COX-2.

### In vitro inhibitory activities of the isolated compounds (C1, C2, C4, and C5) against TNF-R2, NF-кB, NO production

TNF-R2, NF-_*K*_ B, and NO were measured by ELISA kit for compounds (**C1**, **C2**, **C4**, **C5**). The 50% inhibitory concentration (IC_50_) values were calculated from the concentration-inhibition response curve.

**C5** (IC_50_ 5.17 ± 0.07 ng/mL), exhibited higher binding affinity to TNF-R2 (TNF-Receptors) than certolizumab with IC_50_ (6.70 ± 0.07 ng/mL) at (*p* < 0.05). (Table 1). Concerning inhibition of NF-_K_B, **C5** had a significantly lower IC_50_ of 6.07 ± 0.07 Pg/mL than curcumin standard (IC_50_ 7.80 ± 0.03 Pg/mL) at (*p* < 0.05). Moreover, **C1**, **C2**, and **C4** showed significantly moderate suppression of NF-_K_B (Table 1). These results were in accordance with Lee et al.^21^**,** reporting that 7-*O*-methyl aromadendrin exhibited anti-inflammatory activity by suppressing the nuclear translocation of NF-_K_B. A previous study showed that sakuranetin could treat inflammation through the reduction of NF-_K_B^14^ while (*E*)-cinnamic acid showed moderate suppression of NF-_K_B and TNF-R2^22^. Compared to standard 7-nitroindazole, nitric oxide synthase inhibitor^23^ (IC_50_ = 49.2 ± 0.80 µg/mL), **C5** significantly inhibited NO with IC_50_ of 38 ± 0.04 µg/mL at (*p* < 0.05). (Table 1).

### Molecular docking

**Figure 2:**
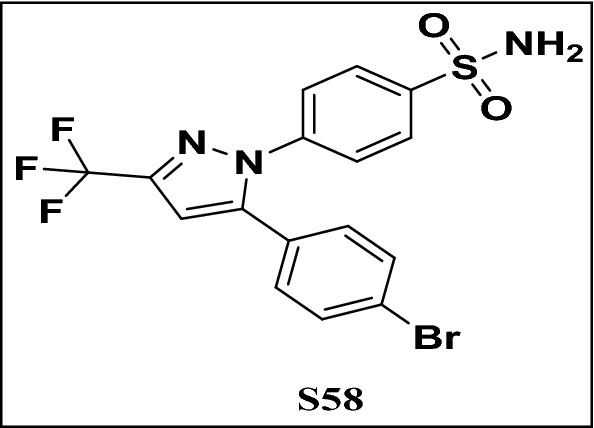
Co-crystallized ligand S58 [4-(5-(4-bromophenyl)-3-(trifluoromethyl)-*1H*-pyrazol-1yl) benzenesulfonamide].

Based on the previous promising in vitro biological evaluation results, compounds (**C1, C2, C4,** and **C5**) isolated from MCF were selected for molecular docking studies into the binding site of COX-2 enzyme to develop an insight into the putative intermolecular interactions and explore the possible binding pattern behind the inhibitory activities of these compounds. The choice of COX-2 for performing the docking study was mainly because it is a vital key enzyme in inflammation and is considered a rate-limiting enzyme that catalyzes prostaglandin production, responsible for the formation of inflammatory mediators^24–28^. The docking study was performed using Molecular Operating Environment software (MOE, 2016.0802). The X-ray crystal structures of COX-2 (PDB ID: 1CX2) with its co-crystallized ligand S58 [4-(5-(4-bromophenyl)-3-(trifluoromethyl)-*1H*-pyrazol-1yl) benzene sulfonamide] were obtained from the Protein Data Bank (PDB) (Fig. 2).

The docking poses were chosen based on the top-scored conformation with the optimum binding interactions found by the MOE search algorithm and scoring function. Binding affinities to the binding pockets enzyme were also determined by binding energy scores, the creation of binding interactions with neighboring amino acid residues, and the relative alignment of docked poses in relation to co-crystallized ligands.

**Figure 3:**
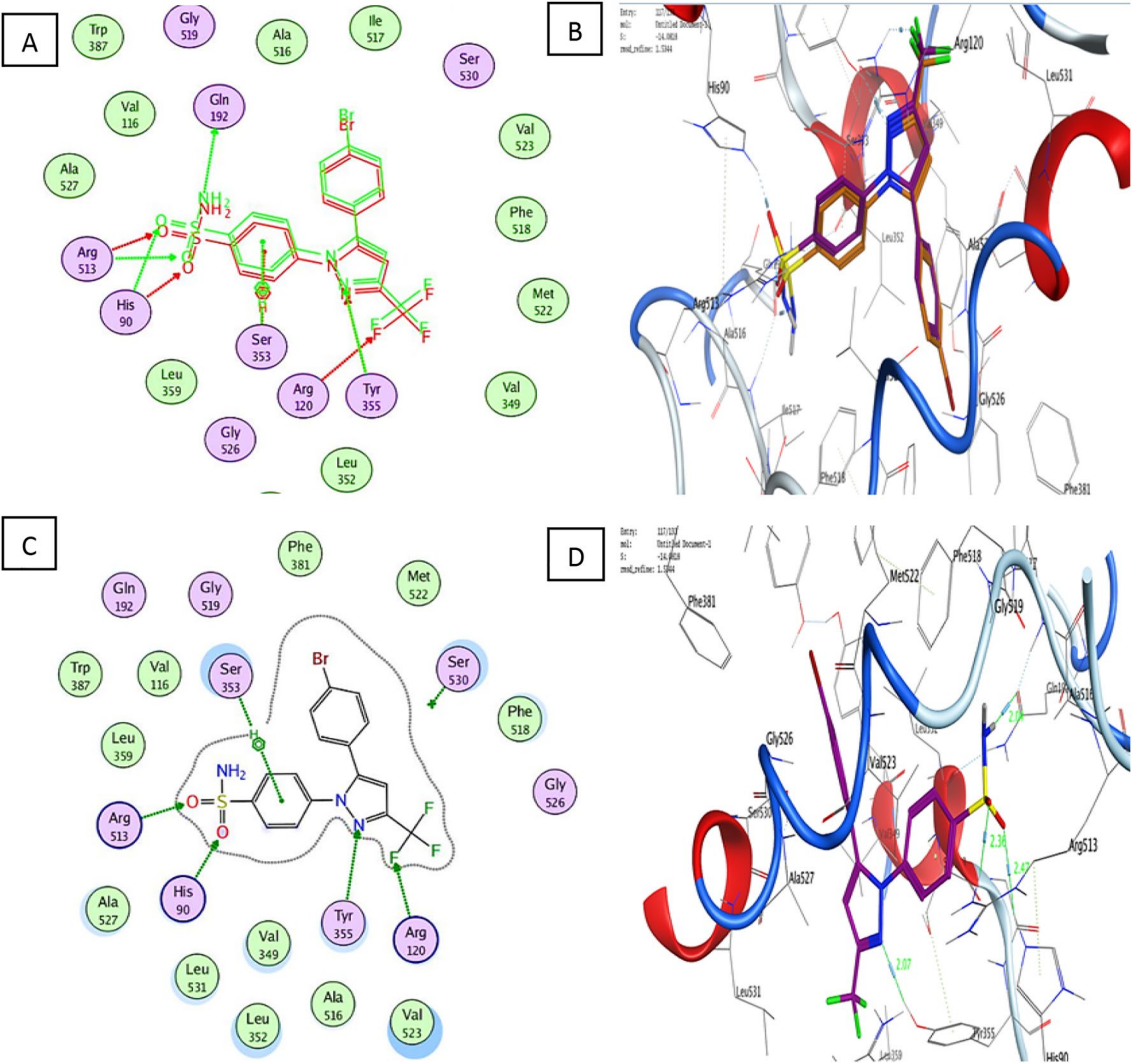
An overlay and binding pattern of S58 into COX-2 active site (PDB 1CX2) 2D (**A**,**C**) 3D (**B**,**D**) co-crystalized ligand (Brown), S58 (Purple).

The proposed docking algorithm was validated by re-docking the co-crystallized ligands S58 into the binding site. The initial poses generated from the PDB were retrieved with a root mean square deviation (RMSD) of 1.53 Å and a docking score of − 14.08 kcal/mol, for COX-2 (Fig. 3). These results indicated that the docking protocol could reliably predict docking poses for the tested compounds. It was reported that values less than 1.5 or 2 Å were indicators of a successful and reliable docking protocol^29^.

### Docking to COX-2 active site

**Figure 4:**
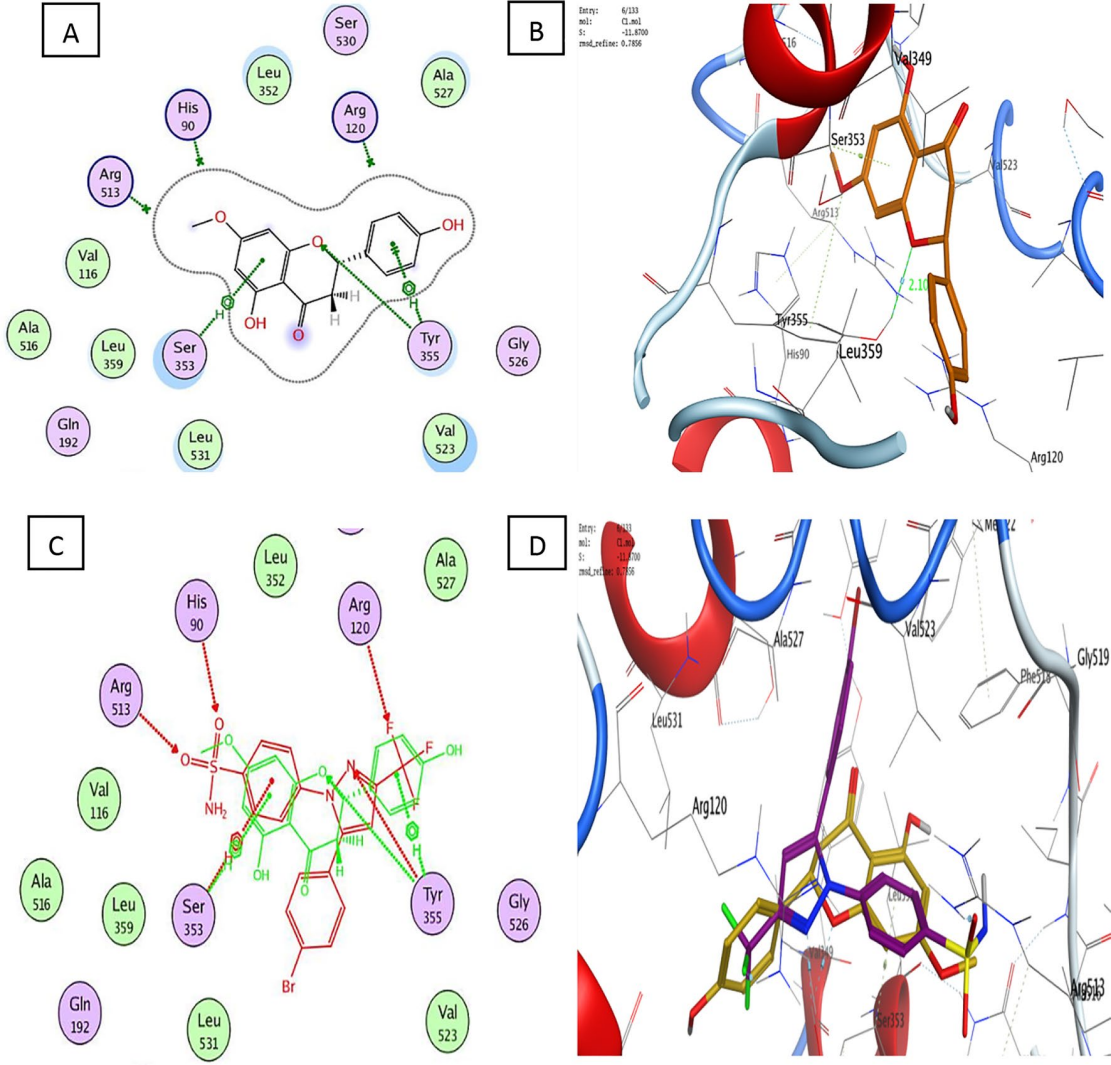

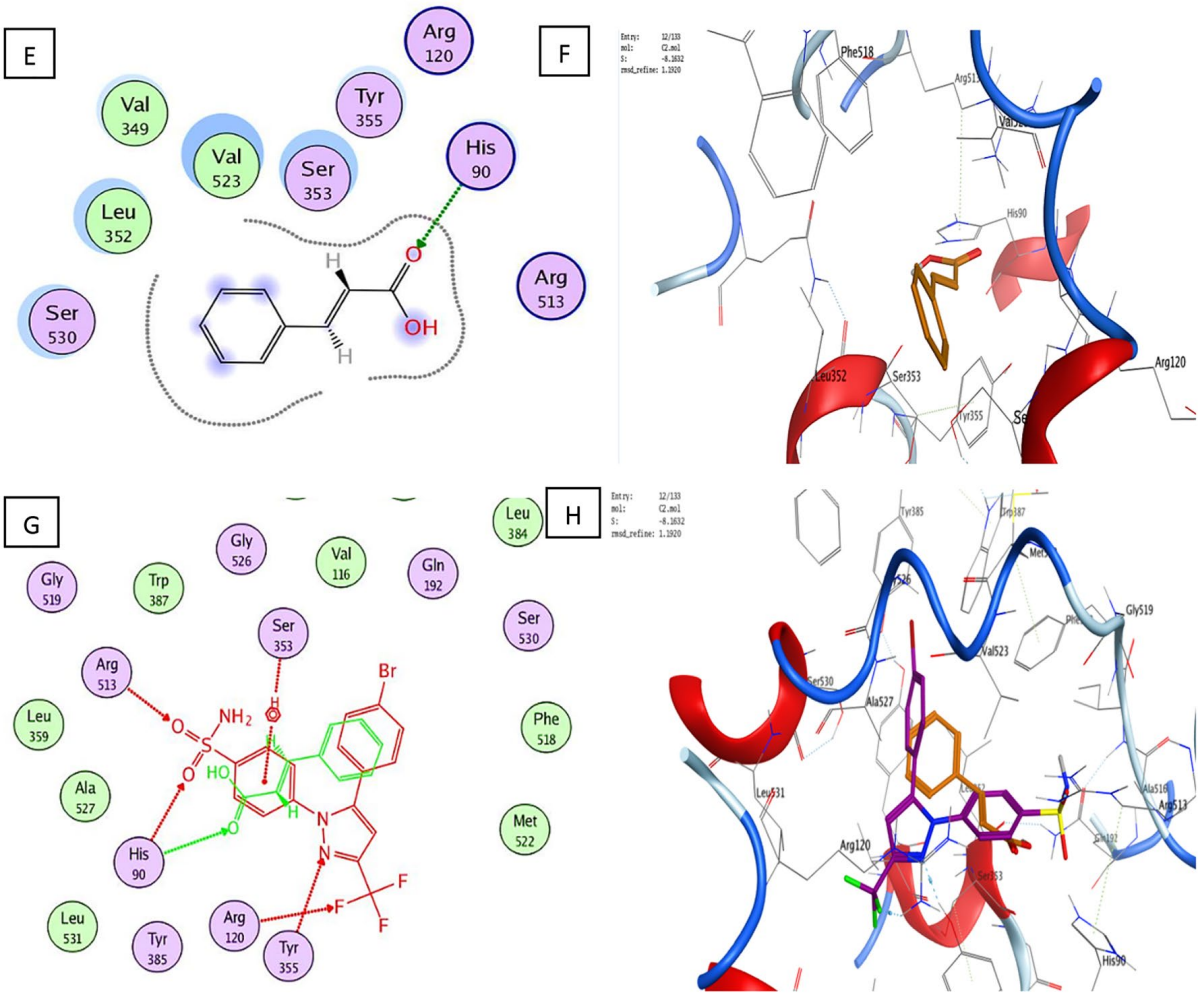

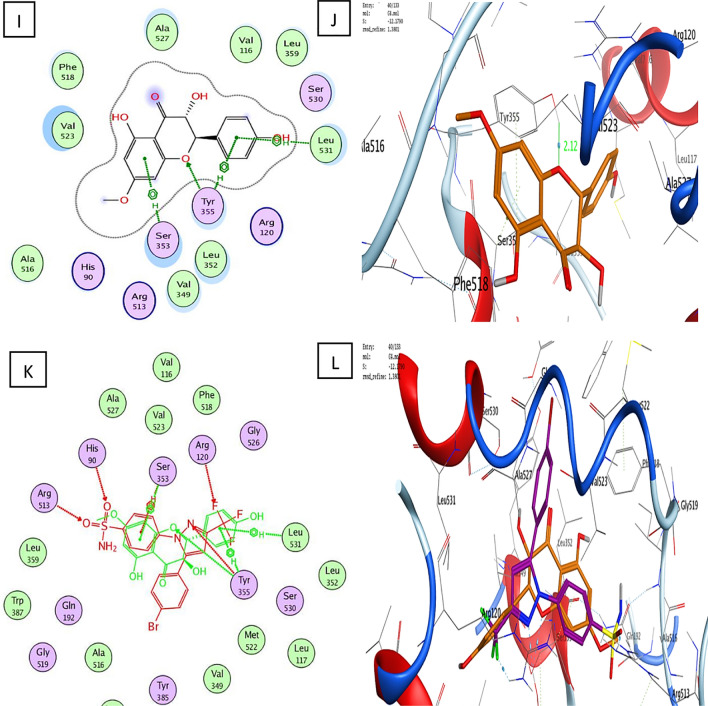

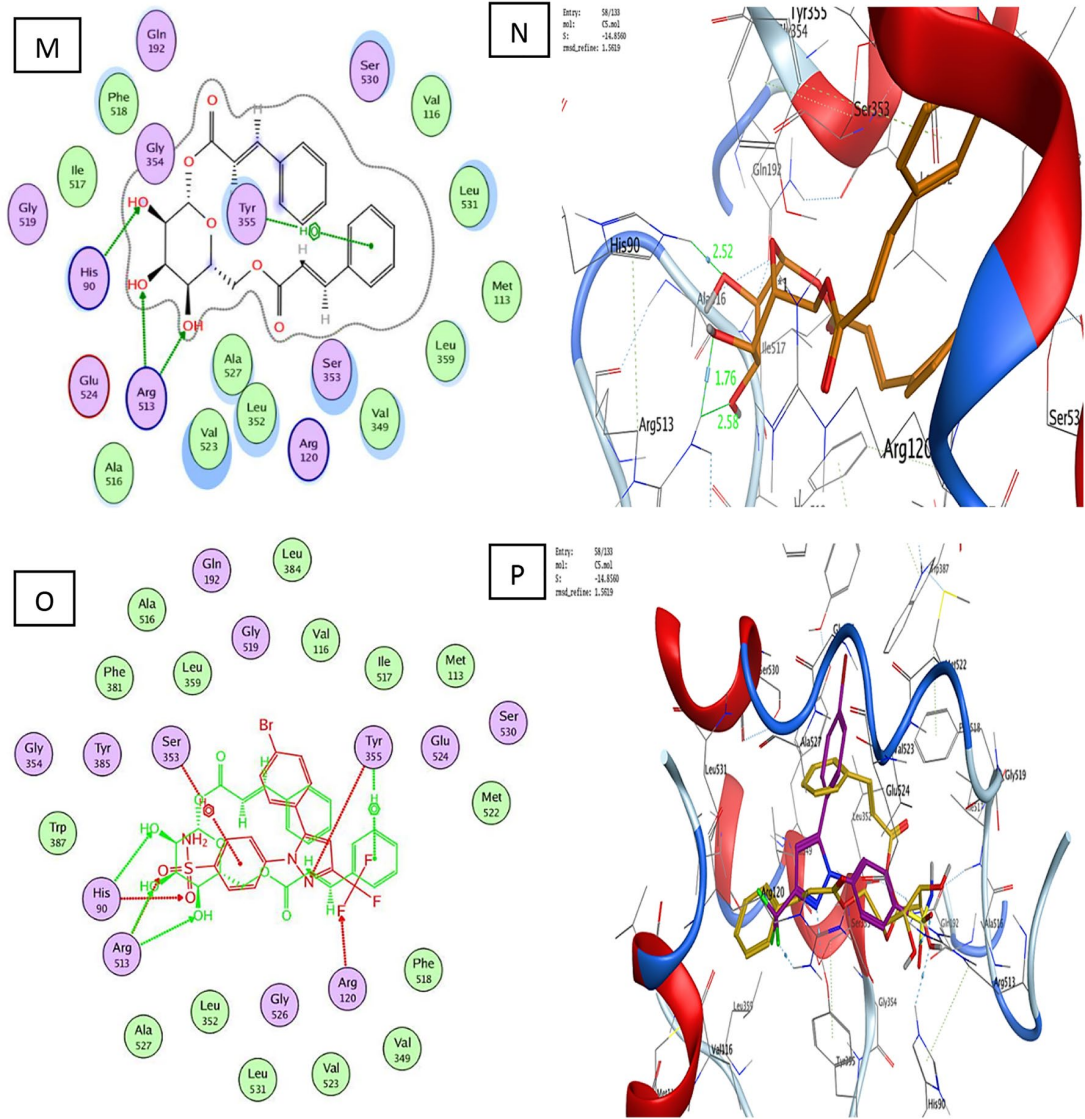
Docking and binding pattern into COX-2 active site (PDB 1CX2) of Target compound C1, 2D (**A**), 3D (**B**), an overlay of the docked pose of compound C1 with co-crystalized ligand S58 2D (**C**), 3D (D) S58 (purple), C1 (yellow). Regarding compound C2, 2D (**E**), 3D (**F**), an overlay of the docked pose of compound C2 with co-crystalized ligand S58 2D (**G**), 3D (**H**) S58 (purple), C2 (yellow). Concerning C4, 2D (**I**), 3D (**J**), an overlay of the docked pose of compound C4 with co-crystalized ligand S58 2D (**K**), 3D (**L**) S58 (purple), C4 (yellow). Finally target compound C5, 2D (**M**), 3D (**N**), an overlay of the docked pose of compound C5 with co-crystalized ligand S58 2D (**O**), 3D (**P**) S58 (purple), C5 (yellow).

With a binding energy score (S) of − 14.08 kcal/mol and (RMSD) of 1.53, the co-crystallized ligand S58 bound to the active site of the COX-2 enzyme displayed two hydrogen bonds of 3.46 and 3.40 Å between the sulfonamide oxygen and His90 and Arg513, respectively. Furthermore, two hydrogen bonds of 3.03 and 3.36 Å were observed between pyrazole nitrogen and the trifluoromethyl group with Tyr355 and Arg120, respectively. Another hydrophobic contact of 3.85 Å formed between the sulfonamide phenyl ring and Ser353 (Fig. 3A–D). Molecular docking studies of the target (**C1**) with a binding energy score (S) of − 11.87 kcal/mol and (RMSD) of 0.78 indicated that the 4-hydroxyphenyl ring and the chroman aromatic ring form two hydrophobic interactions of 4.24 and 3.82 Å with Ser353 and Tyr355, respectively. Also, the hydroxyl group forms a hydrogen bond of 2.91 Å with Tyr355 (Fig. 4A,B). Moreover, the orientation of (**C1**) in the binding pocket was quite similar to that of the native ligand (Fig. 4C,D). Molecular docking studies of the target (**C2**) with a binding energy score (S) of − 8.16 and (RMSD) of 1.19 revealed that the (*E*)-cinnamic acid carbonyl group participated in H-bonding of 3.43 Å with His90 (Fig. 4E,F). Furthermore, the orientations of **(C2)** and the co-crystalized ligand S58 inside the binding pocket of COX-2 were quite similar (Fig. 4G,H**)**. Examination of the best-docked pose of (C4) with (S) of − 12.17 and (RMSD) of 1.38 demonstrated that it was perfectly positioned in the active site of the COX-2 enzyme. It was lodged in the active site through a hydrogen bond of 2.93 Å between chroman ring oxygen and hydrogen bond donor Tyr355. In addition, two hydrophobic interactions between the 4-hydroxy phenyl ring of 4.24 and 4.54 Å with Tyr355 and Lue 4.53, respectively. On the same track, chroman aromatic rings form hydrophobic interaction of 3.81 Å with Ser353 (Fig. 4I,J). Interestingly, their orientation in the binding pocket was quite similar to that of the co-crystallized ligand **S58**, occupying the same position and spatial area as depicted in (Fig. 4 K,L). With regard to (C5), the best-docked position is being examined with (S) of − 14.85 and (RMSD) of 1.56. Three significant H-bonds were observed between the Arg513 and His90 and the 3,4,5-trihydroxytetrahydro-2H-pyrans of 2.56, 3.55, and 3.43 Å, respectively. The complex formed was further stabilized by the hydrophobic interaction of 4.71 Å between the cinnamate aromatic ring and Tyr355 (Fig. 4M,N). Interestingly, its placement in the binding pocket was very similar to that of the co-crystallized ligand S58 in terms of position and spatial orientation, as depicted in (Fig. 4O,P) (Table 2).

**Table 2:** The molecular docking results.

Ligand/compounds	Binding energy score (S) (Kcal/mol)	RMSD	Binding distance (Å)	Type of binding interactions	Residues involved in the interaction
S58	− 14.08	1.53	3.46 and 3.40	Hydrogen bonds	The sulfonamide oxygen with His90 and Arg513
			3.03 and 3.36	Hydrogen bonds	Pyrazole nitrogen and the trifluoromethyl group with Tyr355 and Arg120
			3.85	Hydrophobic interaction	Sulfonamide phenyl ring with Ser353
C1	− 11.87	0.78	4.24 and 3.82	Hydrogen bonds	4-Hydroxyphenyl ring and chroman aromatic ring with Ser353 and Tyr355
			2.91	Hydrogen bond	Phenolic hydroxyl group with Tyr355
C2	− 8.16	1.19	3.43	Hydrogen bond	Carbonyl group with His90
C4	− 12.17	1.38	2.93	Hydrogen bond	Chroman ring oxygen with Tyr355
			4.24 and 4.54	Hydrogen bonds	4-Hydroxy phenyl ring with Tyr355 and Lue4.53
			3.81	Hydrogen bond	Chroman aromatic ring with Ser353
C5	− 14.85	1.56	2.56, 3.55, and 3.43	Hydrogen bonds	3,4,5-trihydroxytetrahydro-2H-pyran with Arg513 and His90
			4.71	Hydrogen bond	Cinnamate aromatic ring with Tyr355

Based on earlier docking experiments, it is possible to explain why compound (C2) has the lowest in vitro activity and why compounds (C5), (C4), and (C1) have higher in vitro activity. Compound C5 displayed the most effective through non-covalent bonding interactions with three essential amino acids Arg513, His90, and Tyr355, as does the cocrystallized ligand S58, whereas C4 exhibits less activity than (C5) because it interacts with only two amino acids: Ser353 and Tyr355. Additionally, (C4) has an advantage over (C1) since it can interact with Ser353, Tyr355, and Leu531, but (C1) lacks the interaction with Leu531 and forms interaction only with Ser353 and Tyr355. Furthermore, the least active one, (C2), only interacts with His90, so minimal activity is predictable.

Further, an in silico study was performed in order to predict the pharmacokinetics and toxicity of selected isolated compounds using the Graph-Based Signatures (pkCSM) program^30^ (Table 3). The outcomes for intestinal absorption showed high levels for each isolated compound tested. Additionally, C5 is sparingly soluble in water, whereas the other separated compounds have considerable water solubility. In terms of distribution, C2 and C5 exhibit good volumes of distribution compared with C1 and C4. Concerning metabolism, all compounds inhibited the majority of CYP450 isoenzymes. With respect to excretion, an acceptable total clearance was achieved by C2 and C5. Finally, compounds C2, C4, and C5 are free from toxicity (Table 3).

**Table 3:** Pharmacokinetic and toxicity properties of selected isolated compounds.

Property	Model name [unit]	Predicted value			
		C1	C2	C4	C5
Absorption	Water solubility [numeric (log mol/L)]	− 3.198	− 2.163	− 3.224	− 4.34
	Caco2 permeability [numeric (log Papp in 10^–6^ cm/s)]	1.035	1.696	− 0.282	0.857
	Intestinal absorption (human) [Numeric (% Absorbed)]	92.308	98.862	81.667	69.774
	Skin permeability [numeric (log Kp)]	− 2.971	− 2.652	− 2.778	− 2.951
	P-glycoprotein substrate [categorical (yes/no)]	Yes	No	Yes	Yes
	P-glycoprotein I inhibitor [categorical (yes/no)]	No	No	No	Yes
	P-glycoprotein II inhibitor [categorical (yes/no)]	No	No	No	Yes
Distribution	VDss (human) [numeric (log L/kg)]	0.013	− 1.017	0.337	− 0.926
	Fraction unbound (human) [numeric (Fu)]	0.202	0.395	0.191	0.048
	BBB permeability [numeric (log BB)]	− 0.191	0.215	− 1.043	− 1.04
	CNS permeability [ Numeric (log PS)]	-2.221	− 1.89	− 3.182	− 3.4
Metabolism	CYP2D6 substrate [categorical (yes/no)]	No	No	No	No
	CYP3A4 substrate [categorical (yes/no)]	No	No	No	Yes
	CYP1A2 inhibitor [categorical (yes/no)]	Yes	No	No	No
	CYP2C19 inhibitor [categorical (yes/no)]	Yes	No	Yes	No
	CYP2C9 inhibitor [categorical (yes/no)]	Yes	No	No	No
	CYP2D6 inhibitor [categorical (yes/no)]	No	No	No	No
	CYP3A4 inhibitor [categorical (yes/no)]	No	No	Yes	Yes
Excretion	Total clearance [numeric (log ml/min/kg)]	0.136	0.775	0.092	0.392
	Renal OCT2 substrate [categorical (yes/no)]	No	No	No	No
Toxicity	AMES toxicity [categorical (yes/no)]	Yes	No	No	No
	Max. tolerated dose (human) [Numeric (log mg/kg/day)]	0.123	0.462	0.037	− 0.135
	hERG I inhibitor [categorical (yes/no)]	No	No	No	No
	hERG II inhibitor [categorical (yes/no)]	No	No	No	No
	Oral rat acute toxicity (LD50) [numeric (mol/kg)]	2.314	2.311	2.302	2.572
	Oral rat chronic toxicity (LOAEL) [numeric (log mg/kg_bw/day)]	1.331	2.183	1.794	2.448
	Hepatotoxicity [categorical (yes/no)]	No	No	No	No
	Skin sensitisation [categorical (yes/no)]	No	Yes	No	No
	*T. pyriformis* toxicity [numeric (log ug/L)]	0.637	0.248	0.396	0.316
	Minnow toxicity [numeric (log mM)]	0.632	1.427	1.554	0.926

## Conclusion

The current study revealed that *E. maculata* resin is a promising Egyptian natural medicine, rich in phenolics and flavonoids, and could be considered a new therapeutic candidate for inflammation. Moreover, 1,6-Dicinnamoyl-*O-α*-D-glucopyranoside (**C5**) showed the best binding activity towards the COX-2 active site. This binding looks similar to or better than celecoxib which is considered the first drug of choice for COX-2 inhibition, indicating the importance of this compound as a natural product for the treatment of inflammation. Moreover, further biological and clinical studies are required on the standardized bioactive extract; EME to verify its possible use as adjuvant therapy in the management of inflammation and its disorders. The continuous need for new natural therapeutic agents requires many hard efforts to open new opportunities from available traditional medicine resources. This can be reached only by understanding their chemical constituents and their medicinal actions, which might be a hard task as a result of the many hundreds of compounds in each formula^31^. Fortunately, the in silico tools have not only accelerated the novel natural molecules' discovery but also explained new activities for known compounds^32^.

## Methods

### Plant material

The kino resin of *E. maculata,* cultivated in the Zoo Garden, Giza, Egypt was scratched from the stem in April 2019. It was pulverized and placed in sealed bottles. The plant was identified by Dr. M. Gibali, Senior Taxonomist in the Department of Botany, National Research Center, Giza, Egypt and Agriculture Engineer Mrs. Therese Labib, Senior Botanist, Orman botanic garden, Giza, Egypt. Voucher specimens of *E. maculata* (Sp. # EM 2.7.2019) was deposited at the Department of Pharmacognosy, Faculty of Pharmacy, Cairo University, Egypt. The study complies with local, national, and international guidelines and no specific consent was required for the collection of kino resin from the plant.

### Chemicals for phytochemical study

All solvents used were of AR grade. Pre-coated TLC plates of silica gel GF254 (Merck, Germany) were used for TLC analysis. TLC analysis was performed using CHCl_3_-MeOH (9.5:05) as a solvent system (S1) and the plates were observed under UV light (254 and 366 nm). Kaempferol 7-methyl ether was purchased from Aktin Laboratories, Phytochemicals Division of Aktin Chemicals, Inc. (Chengdu, China). Silica gel 60 (mesh size 230–400) and silica gel H for vacuum liquid chromatography (VLC) were purchased from Merck (Germany).

### In vitro anti-inflammatory activity

Cyclooxygenases (COX-1 and COX-2) were measured by an enzyme immunoassay (EIA) kit (Cayman Chemical, Ann Arbor, MI, USA) according to the manufacturer’s instructions. TNF-R2, NF-_*K*_B, and NO were measured by ELISA kit (#79756, BPS Bioscience, USA), (#CSB-EL015761HU, Cusabio, China).

### General experimental procedures

^1^H-NMR and ^13^C-NMR spectra of the isolated compounds were recorded on a Bruker High-Performance Digital FT-NMR-spectrophotometer (Avance III HD), Bremen, Germany. Chemical shift values were recorded in δ ppm. The solvents used are CDCl_3_ and CD_3_OD (Cambridge isotope laboratory, USA). The obtained data were processed using Mestrenova NMR processor software, version 6.0.2-5475.

### Extraction of *E. maculata* resin

The air-dried powdered resin (140 g) of *E. maculata* exudate (EME) was extracted with methanol (3 × 100 mL) using a sonicator (Soltec Co., 230/240 V, 50/60 Hz, Italy) for 15 min. The extract was concentrated under reduced pressure to a constant weight to yield dry methanol residue; (MEEM, 130 g), then it was kept in a desiccator to dry over anhydrous CaCl_2_.

The methanolic extract of *E. maculata* exudate (MEEM, 120 g) was suspended in distilled water (300 mL) and partitioned with CH_2_Cl_2_ (4 × 100 mL) at room temperature to give methylene chloride fraction (MCF, 7 g) on evaporation.

### Isolation of the major compounds from methylene chloride fraction

The MCF (7 g) was dissolved in the least volume of methanol, mixed well with about 20 g of silica gel 60 and left at room temperature to dry, then applied onto the top of a VLC packed to a glass column with silica gel H (15 × 6 cm i.d., 210 g). Gradient elution with increasing polarity (5% increments) was carried out starting with 100% *n*-hexane, *n*-hexane/CH_2_Cl_2_, CH_2_Cl_2_, and finally CH_2_Cl_2_/EtOAc mixture, up to 100% EtOAc and finally washing with 100% methanol. Fractions, 200 mL each, were separately concentrated and monitored by TLC. The developed plates were sprayed with *p*-anisaldehyde/H_2_SO_4_ spray reagent and then heated at 115 °C. Similar fractions were pooled together. Different collective fractions were subjected to chromatographic separation and purification techniques to obtain five compounds (**C1**–**C5**)**.** Fraction I (125 mg; 90–95% CH_2_Cl_2_ in *n*-hexane). It was further purified on Si gel 60 column (15 × 1.5 cm) using *n*-hexane–ethyl acetate (9.5:0.5, v/v) to give yellowish white powder of compound **C1** (70 mg). Fraction II (140 mg; 95% CH_2_Cl_2_ in EtOAc) showed two major spots at R_*ƒ*_ = 0.56 and 0.43, respectively (TLC, CH_2_Cl_2_-MeOH; 9.5:0.5). It was subjected to further purification on another Si gel 60 column (25 × 1.5 cm) using CH_2_Cl_2_-MeOH (9.7:0.3 v/v) to give compound **C1** (25 mg) and compound **C2** as a white amorphous powder (40 mg). Fraction III (1.2 g; 85% CH_2_Cl_2_ in EtOAc) was further purified on Si gel 60 column (35 × 3 cm) using CH_2_Cl_2_-MeOH (9:1, v/v) to give subfraction (440 mg) that showed one major spot at R_ƒ_ = 0.39 (TLC, CH_2_Cl_2_-MeOH; 9:1). It was subjected to further purification on Si gel 60 column (25 × 1.5 cm, 230–400 mesh) using CH_2_Cl_2_/MeOH (9.5:0.5, v/v) to give yellow powder of compound **C3** (30 mg).

Fraction IV (1.2 g; 80% CH_2_Cl_2_ in EtOAc) showed one major spot at R_ƒ_ = 0.44 (TLC, CH_2_Cl_2_-MeOH; 9:1). It was further purified on another Si gel 60 column (35 × 3 cm) using CH_2_Cl_2_-MeOH (9.9: 0.1, v/v) to give yellowish white powder of compound **C4** (300 mg). Fraction V (100 mg; 40% CH_2_Cl_2_ in EtOAc) showed one major spot at R_ƒ_ = 0.58 (TLC, CH_2_Cl_2_-MeOH; 9.5:0.5). It was further purified on another Si gel 60 column (15 × 1.5 cm) column using CH_2_Cl_2_-MeOH (9.9:0.1, v/v) to give white powder of compound **C5** (45 mg).

### ^1^H NMR and ^13^C-NMR spectra of the isolated compounds

**Compound C1** (70 mg), was obtained as yellowish white powder, with *R*_*f*_ 0.78 (S1), and it was identified as **sakuranetin **^9,15^**.**

^**1**^**H NMR (400 MHz, CDCl**_**3**_**)** δ_H_ 12.00 (s, OH), 7.26 (2H, d, *J* = 8.2 Hz, H-2', H-6'), 6.81 (2H, d, *J* = 8.3 Hz, H-3', H-5'), 5.99 (2H, d, *J* = 2.2 Hz, H-6, H-8), 5.28 (1H, dd, *J* = 13.0, 2.8 Hz, H-2), 3.73 (s, 3H, OMe), 3.02 (1H, dd, *J* = 17.2, 13.0 Hz, H_3ax_), 2.71 (1H, dd, *J* = 17.2, 2.9 Hz, H_3eq_). ^**13**^**C-NMR (100 MHz, CDCl**_**3**_**):** δc 80.9 (C-2), 44.6 (C-3), 198.1(C-4), 165.3 (C-5), 96.3 (C-6), 169.6 (C-7), 95.5 (C-8), 164.7 (C-9), 104.4 (C-10), 56.9 (OCH3), 129.3 (C-1'), 130.9 (C-2'), 116.8 (C-3'), 159.1 (C-4'), 116.8 (C-5'), 129.3 (C-6').

**Compound C2 (40 mg),** was isolated as white amorphous powder**,** with *R*_*f*_ 0.56 (S1), and it was identified as ***Trans*- cinnamic acid **^9,16^**.**

^**1**^**H-NMR (400 MHz, MeOH-d**_**4**_**)** δ_H_ 7.69 (1H, d, *J* = 16 Hz) and 6.5 (1H, d, *J* = 16 Hz) that could be attributed to *trans* olefinic protons of *α, β* unsaturated ketone attached to C-1, 7.43 (2H, m, H2/6) and 7.62–7.6 (3H, m, H 3, 4, 5).

**Compound C3** (30 mg), was obtained as yellow powder, with *R*_*f*_ 0.53 (S1), and it was identified as **Kaempferol -7- methyl ether **^15,17^.

^**1**^**H-NMR (400 MHz, MeOH-d**_**4**_**)** δ_H_ 6.33 (1H, d, *J* = 2.1 Hz, H-6), 6.62 (1H, *d*, *J* = 2.1 Hz, H-8), 8.14 (2H, d, *J* = 8.9 Hz, H-2'/6'), 6.93 (2H, d, *J* = 8.9 Hz, H-3'/5'), 3.91 (3H, s, Me-7). ^**13**^**C-NMR (100 MHz, MeOH-d**_**4**_**):** δc 146.93 (C-2), 135.93 (C-3), 176.08 (C-4), 160.01 (C-5), 97.46 (C-6), 165.98 (C-7), 91.43 (C-8), 159.35 (C-9), 104.47 (C-10), 55.03 (OCH3), 122.24 (C-1'), 129.93 (C-2', 6'), 115.24 (C-3',5'), 156.64 (C-4').

**Compound C4** (30 mg), was isolated as yellowish white powder, with *R*_*f*_ 0.49 (S1), and it was identified as **7-*O*-methyl aromadendrin **^9,15^.

^**1**^**H-NMR (400 MHz, MeOH-d**_**4**_**)** δ_H_ 5.02 (1H, d, *J* = 11.5 Hz, H-2), 4.59 (1H, d, *J* = 11.5 Hz, H-3), 6.05 (1H, d, *J* = 2.2 Hz, H-6), 6.09 (1H, d, *J* = 2.2 Hz, H-8), 7.37 (1H, d, *J* = 8.5 Hz, H-2'/6'), 6.85 (1H, d, *J* = 8.6 Hz, H-3'/5'), 3.82 (3H, s, Me-7).

**Compound C5 (**25 mg), was obtained as white powder, *R*_*f*_ 0.43 (S1), and it was identified as **1,6-dicinnamoyl-*O-α*-D-glucopyranoside**^9^.

#### ^1^H-NMR (500 MHz, CHCl_3_- d)

#### Cinnamoyl moieties

δ_H_ 7.8, 7.71 (2H, d, *J* = 16, 2*H*α*), 7.53 (4H, m, 2*H2, 2*H6), 7.38 (6H, m, 2*H-3, H4, H5), 6.49 (2H, d, *J* = 16, 2H*β*).

#### *α*-glucose

δ_H_ 5.69 (1H,d, *J* = 3.9), 4.54 (1H, d, *J* = 12, H6ʹ_a_), 4.43 (1H, dd, *J* = 12, 4.5, H6ʹ_b_), 3.74 (1H, m, H-5ʹ), 3.57 (2H, m, H-2ʹ, H-3ʹ), 3.5 (1H, m, H-4ʹ).

#### ^13^C-NMR (125 MHz, CHCl_3_-d)

#### Cinnamoyl moieties

δ_C_ 165.47, 167.23 (2xC = O), 146.46, 145.37 (2xC-*α*), 133.3, 133.87 (2xC-1), 130.39, 130.15 (2xC-3), 128.59 (2x*C-4), 128.54 (2xC-5), 127.89 (2xC-2), 127.82 (2xC-6), 117.05, 116.56 (2xC-*β*).

#### *α*-glucose

δ_C_ 94.03 (C-1), 72.14 (C-2), 76.2 (C-3), 69.43 (C-4), 74.57 (C-5), 63.16 (C-6).

### In silico study for the interaction of the identified compounds

Computer-aided docking experiments were performed using Molecular Operating Environment software (MOE 2016.0802, Chemical Computing Group, Montreal, Canada)^33^. Crystal coordinates from the X-ray crystal structure of COX-2 (PDB ID code: 1CX2, with the inhibitor S58 bound in the active site) were obtained from RCSB protein data bank and processed consequently with the MOE program. Redundant chains, water molecules, and any surfactants were discarded, explicit hydrogen atoms were added to the receptor complex structure and partial charges were calculated. The preparation was completed with a structure preparation module employing protonated 3D function. The co-crystal ligands were extracted from their corresponding proteins and used as reference molecules for the validation study^29^.

The target compounds were constructed using the builder module of MOE. The compounds were then collected in a database and prepared by adding hydrogens, calculating partial charges, and energy minimizing using Force field MMFF94x.

### Docking procedure

The MOE-Site Finder was used to generate the active site of the receptor, and the MOE-Dock was used to dock the ligands within the active site. As a placement method, we used Triangle Matcher, London as a scoring function, and 10 retained poses as parameters. All receptor-ligand complexes were examined further to determine binding interactions and the optimum docking pose. The best-docked complex, which is thought to represent protein–ligand interactions, was chosen based on docking score, ligand alignment at the active site that was similar to the reference ligands, and retention of significant interactions. This docking approach was validated by the successful pose-retrieval of the co-crystal ligand when docked into its corresponding binding site in the crystal. All graphical representations were rendered by MOE. 2016.0802. In silico investigation of Pharmacokinetic and toxicity properties of selected isolated compounds was carried out using the Graph-Based Signatures (pkCSM) program.

### Statistical analysis

Data were analyzed with GraphPad Prism V 6 (GraphPad Software Inc., San Diego, CA, USA). Data were expressed as mean ± standard deviation (SD) and were analyzed using sample t-test using SPSS 26.0 software. Statistical differences yielding p < 0.05 were considered significant.

## Supplementary Information

Supplementary Information.

## Data Availability

All data generated or analyzed during this study are included in this published article and its supplementary information file.
