# Supplementary material for: In silico and in vitro anti-inflammatory study of phenolic compounds isolated from Eucalyptus maculata resin
Source: Sci Rep. 2023 Feb 6;13:2093. doi: 10.1038/s41598-023-28221-y (PMC9902548; doi:10.1038/s41598-023-28221-y)
Supplement: Supplementary file 1 — Supplementary Information. [file 41598_2023_28221_MOESM1_ESM.docx]

**Supplementary materials**

**Journal: Scientific Reports Journal**

***In Silico* and *In Vitro* Anti-inflammatory Study of Phenolic Compounds Isolated from *Eucalyptus maculata* Resin**

Dalia E. Ali^1, ᵟ^, Rania A. El Gedaily^,2, ᵟ^, Shahira M. Ezzat^2,3^, Maged A. El Sawy^4^, Meselhy R. Meselhy^2^, Essam Abdel-Sattar^2, *^

*^1^Department of Pharmacognosy, Faculty of Pharmacy, Pharos University in Alexandria, Alexandria, Egypt.*

*^2^Department of Pharmacognosy, Faculty of Pharmacy, Cairo University, 11562 Cairo, Egypt.*

*^3^Department of Pharmacognosy, Faculty of Pharmacy, October University for Modern Science and Arts (MSA), 6^th^ October, 12451* *Egypt.*

*^4^Department of analytical and pharmaceutical chemistry, Faculty of Pharmacy, Pharos University in Alexandria, Alexandria, Egypt.*

^ᵟ^ Both authors have equal contribution and are considered as first authors

*Corresponding author

Essam Abdel-Sattar

E mail: [essam.abdelsattar@pharma.cu.edu.eg](mailto:essam.abdelsattar@pharma.cu.edu.eg)

Department of Pharmacognosy, Faculty of Pharmacy, Cairo University, El-Kasr El-Aini St, Cairo, 11562, Egypt

Tel: +201065847211


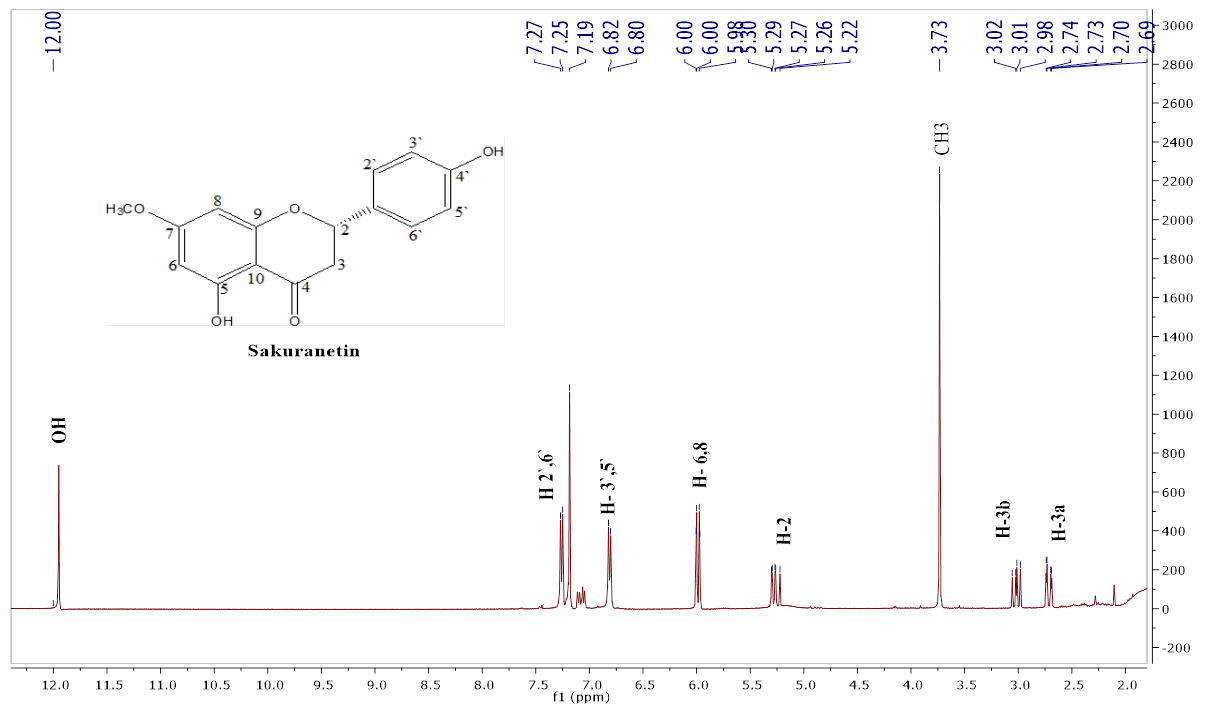


**Fig. 1S: 1H-NMR spectrum of compound C1**

**Fig. 2S: ^1^H-NMR spectrum of compound C2**

**Fig. 3S: ^1^H-NMR spectrum of compound C3**

**Fig. 4S: ^13^C-NMR spectrum of compound C3**

**Fig. 5S: ^1^H-NMR spectrum of compound C4**

**
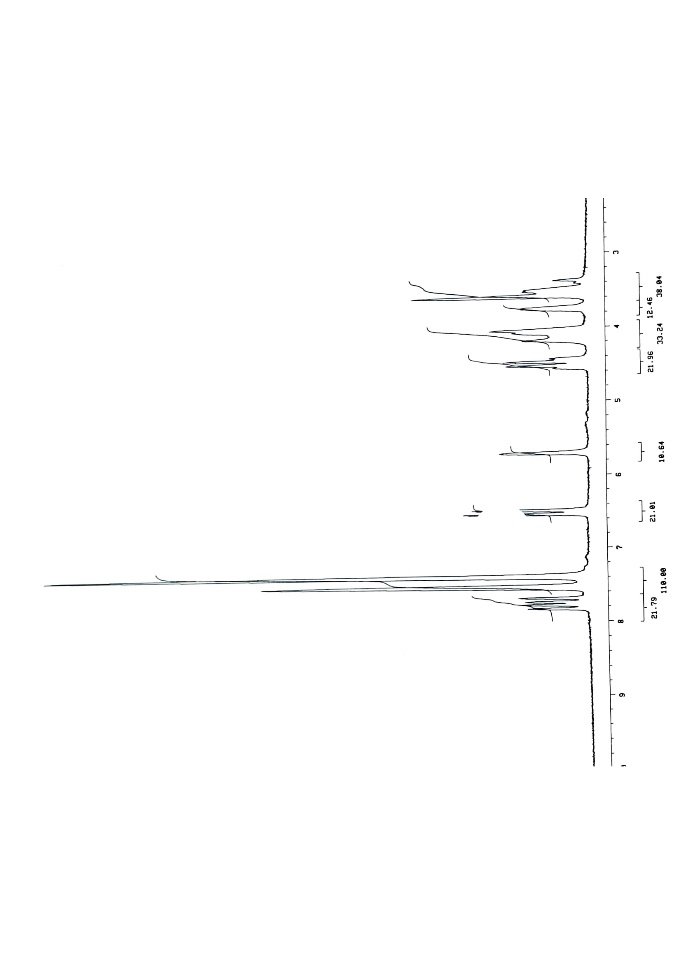
**

**Fig. 6S: ^1^H-NMR spectrum of compound C5**


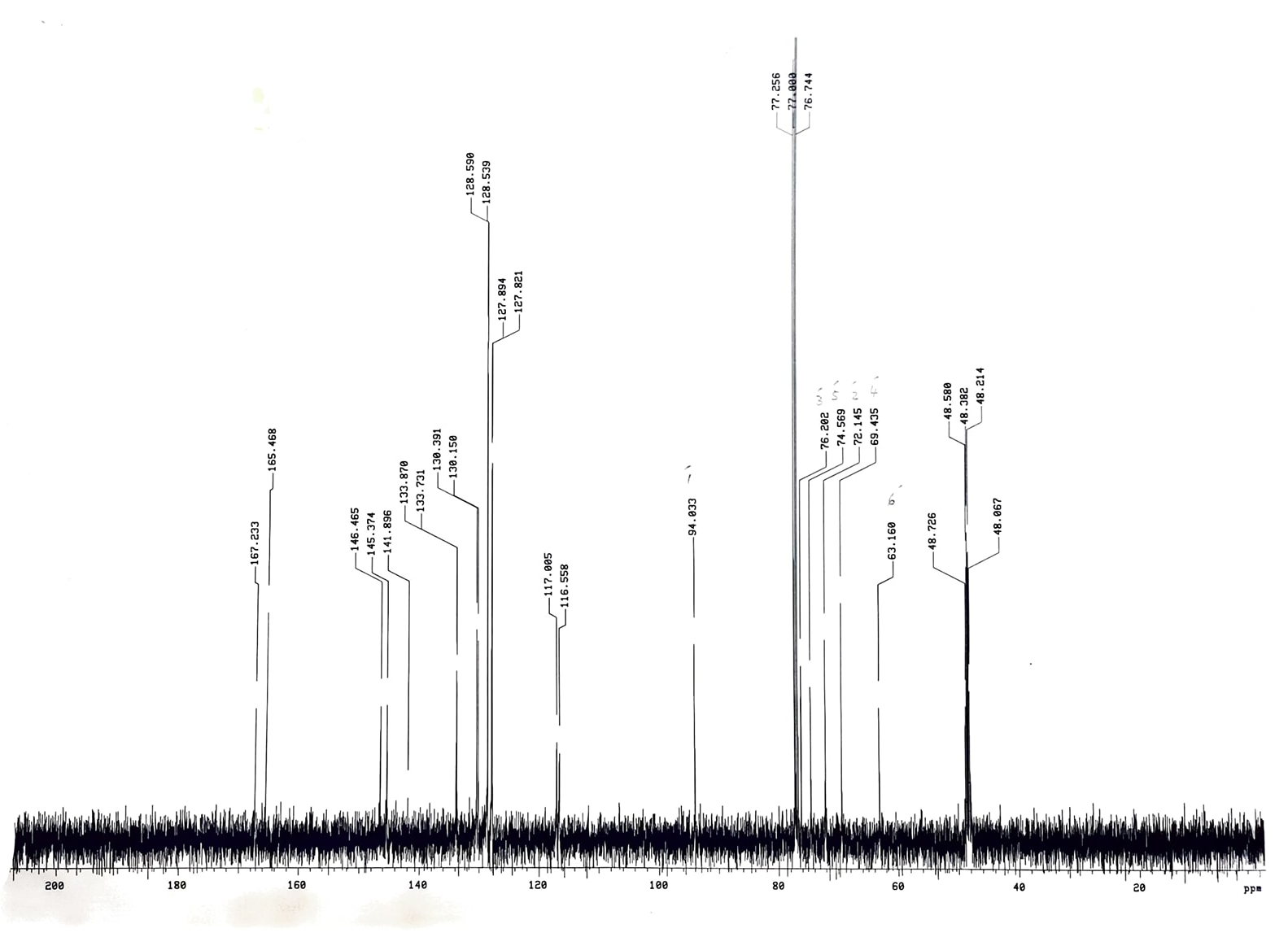


**Fig. 7S: ^13^C-NMR spectrum of compound C_5_**


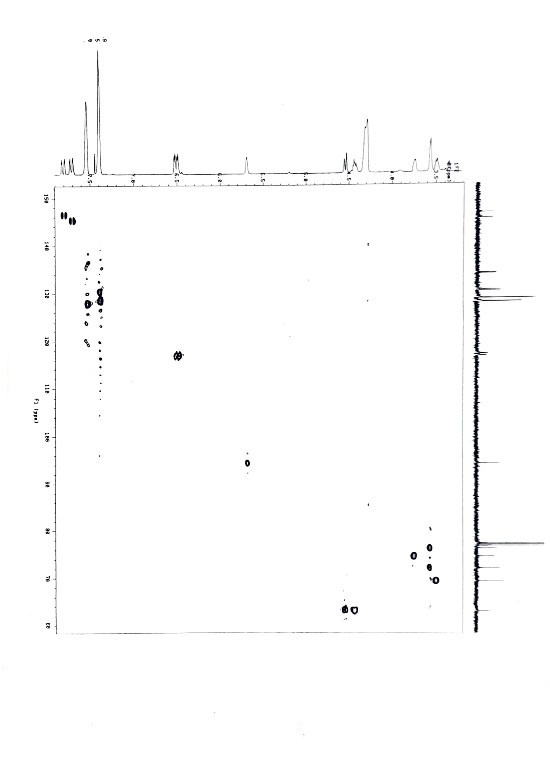


**Fig.8S: HMQC spectrum of compound C5**


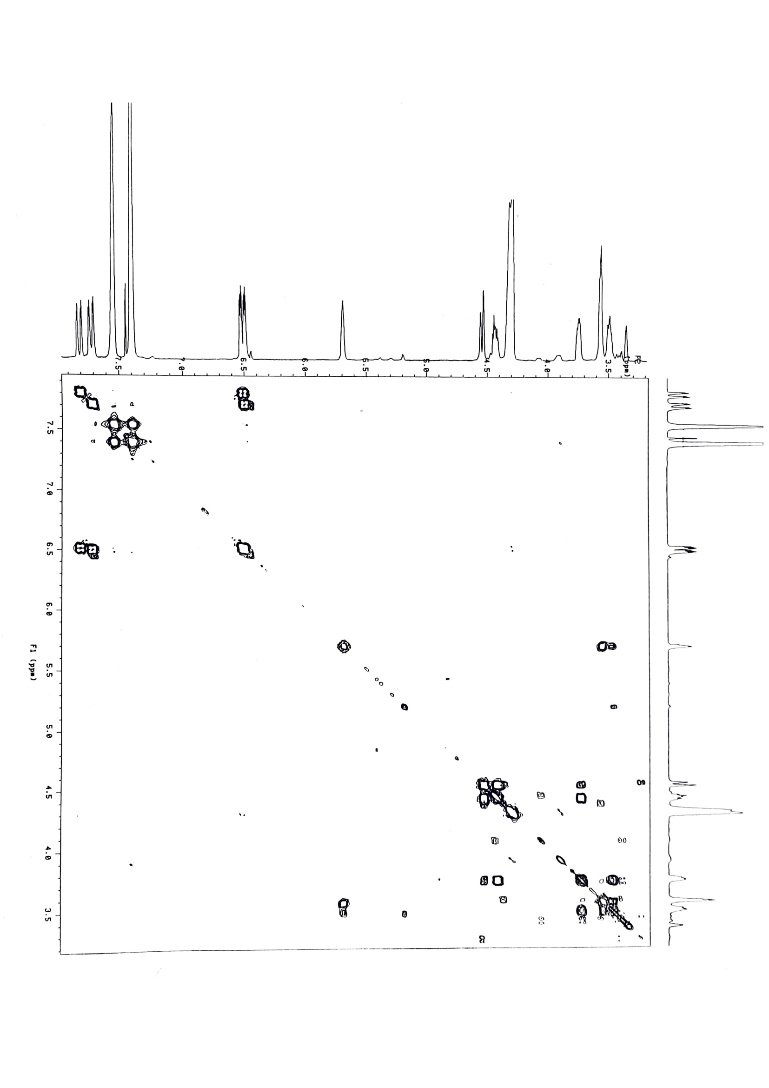


**Fig.9S: H-H COSY spectrum of compound C5**


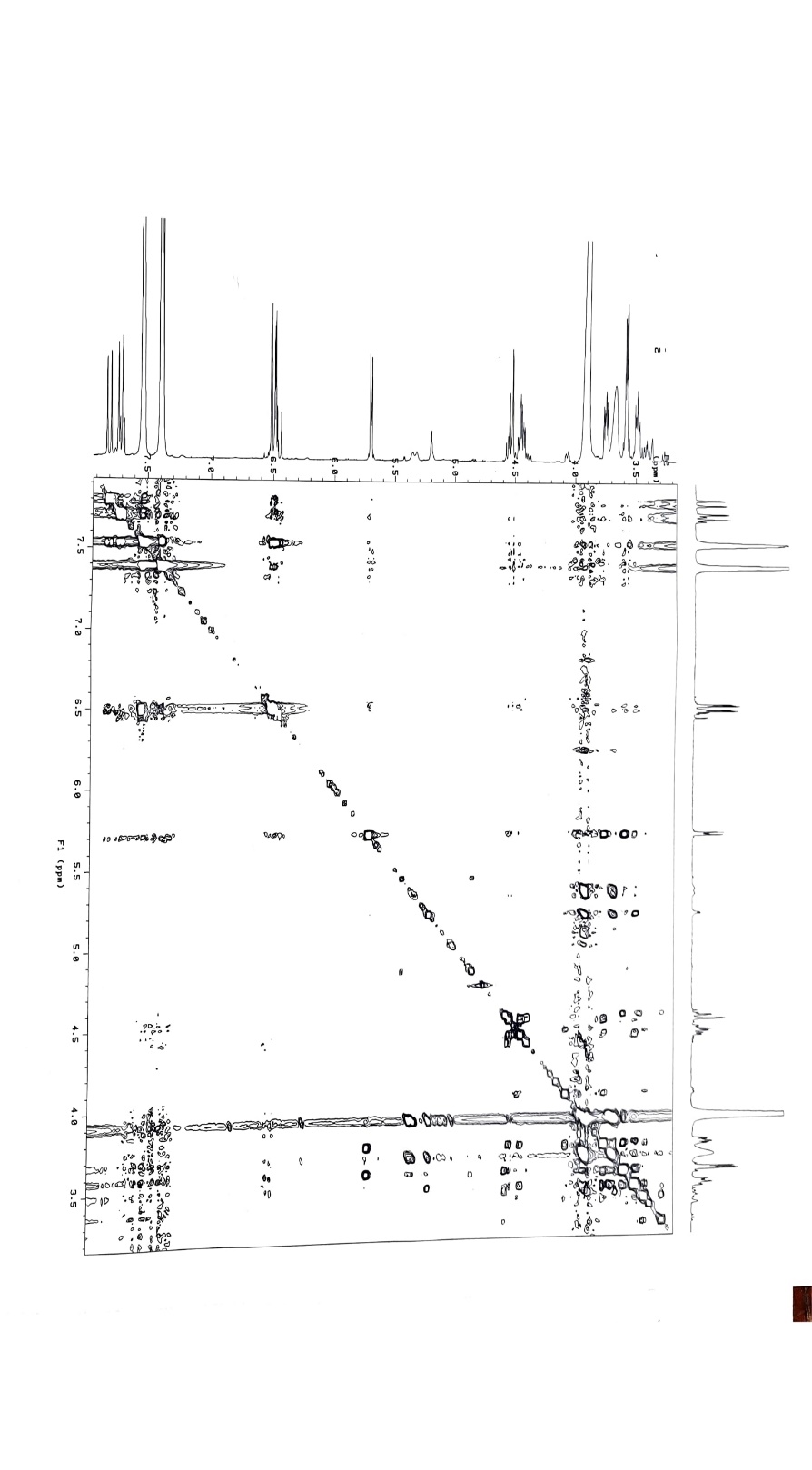


**Fig. 10S: NOESY spectrum of compound C5**


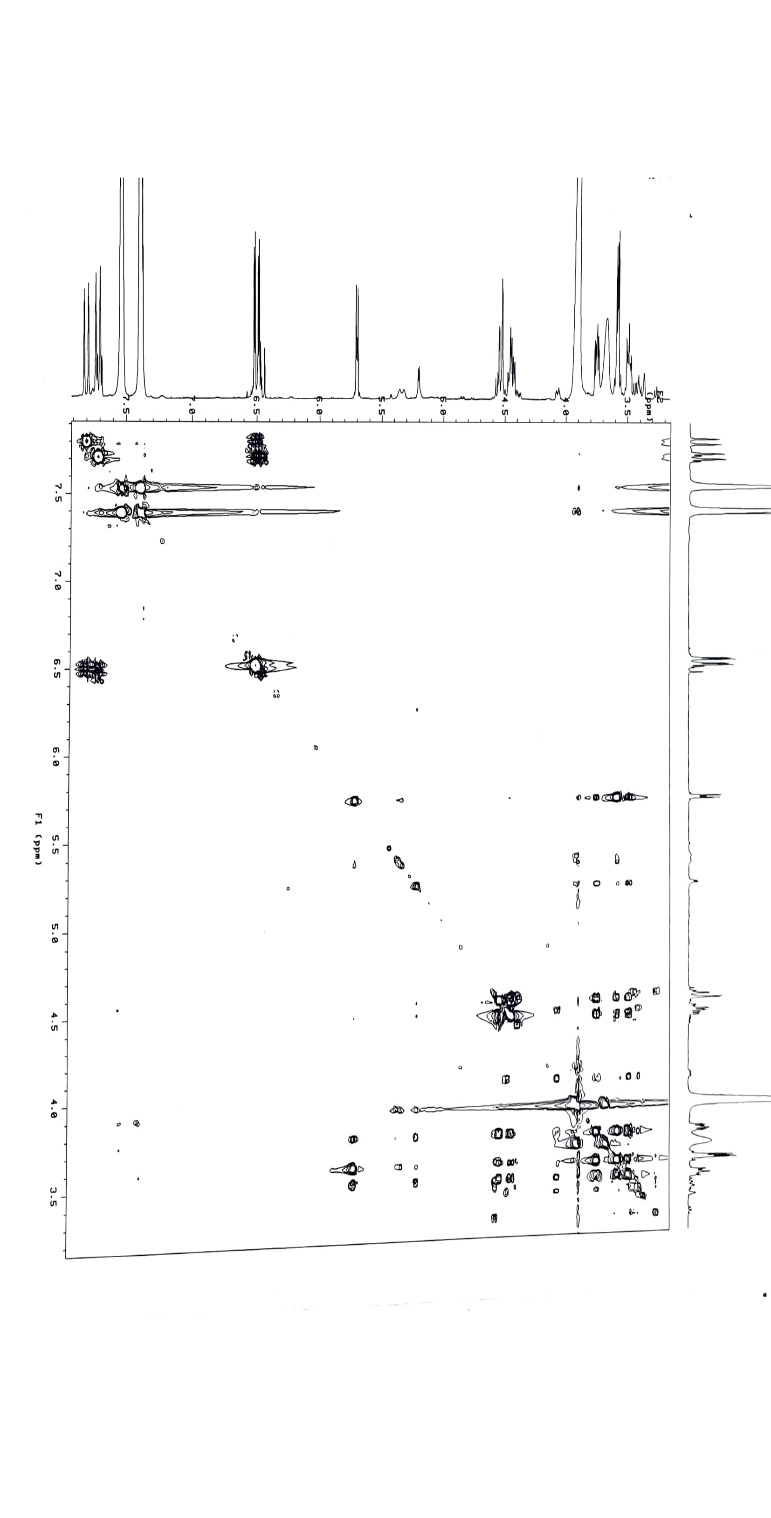


**Fig. 11S: TOCSY spectrum of compound C5**

**References**

1. Abdel-Sattar E, Kohiel M, Shihata I, El-Askary H (2000) Phenolic compounds from Eucalyptus maculata Die Pharmazie 55:623-624.
2. 2. Zhang X et al. (2006) Anti-inflammatory activity of flavonoids fromPopulus davidiana Archives of pharmacal research 29:1102-1108.
3. Ling F, Jiang C, Liu G, Li M, Wang G (2015) Anthelmintic efficacy of cinnamaldehyde and cinnamic acid from cortex cinnamon essential oil against Dactylogyrus intermedius Parasitology 142:1744-1750.
4. Freitas MO, Ponte FA, Lima MAS, Silveira ER (2008) Flavonoids and triterpenes from the nest of the stingless bee Trigona spinipes Journal of the Brazilian Chemical Society 19:532-535
